# Supplementary figures and images for: Population structure of Bathymodiolus manusensis, a deep-sea hydrothermal vent-dependent mussel from Manus Basin, Papua New Guinea
Source: PeerJ. 2017 Aug 21;5:e3655. doi: 10.7717/peerj.3655 (PMC5572536; doi:10.7717/peerj.3655)

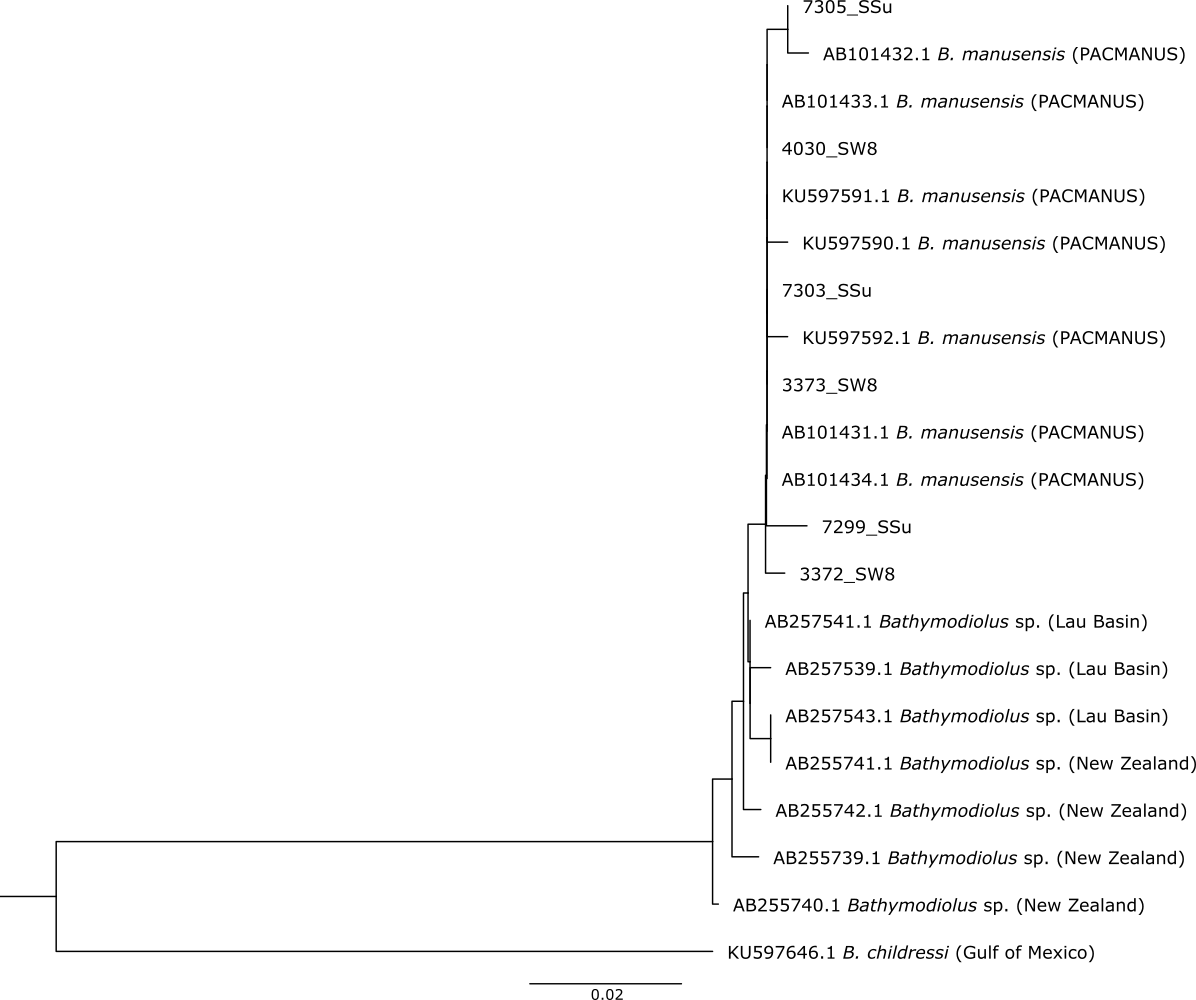

Supplement: Figure S1 — Phylogenetic relationships were inferred using the Neighbor-Joining method (Saitou & Nei, 1987) on 20 Bathymodiolus manusensis sequences from the Western Pacific (370 bp, B. childressi used as outlier). Optimal tree (10,000 bootstrap replicates) with sum branch length of 0.21148514 is shown (Felsenstein, 1985). Evolutionary distances were computed using the Maximum Composite Likelihood method (Tamura, Nei & Kumar, 2004). There were a total of 370 positions in the final dataset. Evolutionary analyses were conducted in MEGA7 (Kumar, Stecher & Tamura, 2016). Felsenstein J. 1985. Confidence limits on phylogenies: an approach using the bootstrap. Evolution 39:783–791 DOI 10.1111/j.1558-5646.1985.tb00420.x. Saitou N, Nei M. 1987. The neighbor-joining method: a new method for reconstructing phylogenetic trees. Molecular Biology and Evolution 4:406–425 DOI 10.1093/oxfordjournals.molbev.a040454. Tamura K, Nei M, Kumar S. 2004. Prospects for inferring very large phylogenies by using the neighbor-joining method. Proceedings of the National Academy of Sciences of the United States of America 101:11030–11035 DOI 10.1073/pnas.0404206101. [file peerj-05-3655-s003.png]
